# Disentangling the Component Processes in Complex Planning Impairments Following Ventromedial Prefrontal Lesions

**DOI:** 10.1523/JNEUROSCI.1814-24.2025

**Published:** 2025-01-31

**Authors:** Eleanor Holton, Bas van Opheusden, Jan Grohn, Harry Ward, John Grogan, Patricia L. Lockwood, Ili Ma, Wei Ji Ma, Sanjay G. Manohar

**Affiliations:** ^1^Department of Experimental Psychology, University of Oxford, Oxford OX2 6GG, United Kingdom; ^2^Imbue, Inc., San Francisco, California 94114; ^3^Wellcome Centre for Integrative Neuroimaging (WIN), University of Oxford, Oxford OX3 9DA, United Kingdom; ^4^Centre for Experimental Medicine and Rheumatology, Queen Mary University London, London E1 4NS, United Kingdom; ^5^Trinity Institute of Neuroscience, Trinity College Dublin, Dublin D02 PX31, Ireland; ^6^Centre for Human Brain Health, Institute for Mental Health and Centre for Developmental Science, School of Psychology, University of Birmingham, Birmingham B15 2TT, United Kingdom; ^7^Department of Developmental and Educational Psychology, Institute of Psychology, Leiden University, Leiden 2300, The Netherlands; ^8^Leiden Institute for Brain and Cognition, Leiden 2333, The Netherlands; ^9^Center for Neural Science and Department of Psychology, New York University, New York 10003; ^10^Nuffield Department of Clinical Neurosciences, University of Oxford, Oxford OX3 7JX, United Kingdom

**Keywords:** cognitive neuroscience, computational modeling, lesions, planning, prefrontal cortex, value-based decision-making

## Abstract

Damage to the ventromedial prefrontal cortex (vmPFC) in humans disrupts planning abilities in naturalistic settings. However, it is unknown which components of planning are affected in these patients, including selecting the relevant information, simulating future states, or evaluating between these states. To address this question, we leveraged computational paradigms to investigate the role of vmPFC in planning, using the board game task “Four-in-a-Row” (18 lesion patients, 9 female; 30 healthy control participants, 16 female) and the simpler “Two-Step” task measuring model-based reasoning (49 lesion patients, 27 female; 20 healthy control participants, 13 female). Damage to vmPFC disrupted performance in Four-in-a-Row compared with both control lesion patients and healthy age-matched controls. We leveraged a computational framework to assess different component processes of planning in Four-in-a-Row and found that impairments following vmPFC damage included shallower planning depth and a tendency to overlook game-relevant features. In the “Two-Step” task, which involves binary choices across a short future horizon, we found little evidence of planning in all groups and no behavioral differences between groups. Complex yet computationally tractable tasks such as “Four-in-a-Row” offer novel opportunities for characterizing neuropsychological planning impairments, which in vmPFC patients we find are associated with oversights and reduced planning depth.

## Significance Statement

The ability to plan in real-world settings is often disrupted after damage to the ventromedial prefrontal cortex (vmPFC). However, naturalistic planning consists of many different cognitive processes, and it is unknown which processes are disturbed by these lesions. Here, we use rich computational models of planning to characterize behavior in two planning tasks performed by patients with vmPFC damage and controls. vmPFC damage only affected behavior in the more complex planning task, and behavioral modeling revealed this was associated with planning less far into the future and overlooking important features. These findings shed light on the neural mechanisms supporting complex planning, demonstrating how novel computational methods can strike the balance between task complexity and interpretability.

## Introduction

Damage to the ventromedial prefrontal cortex (vmPFC) has life-altering effects for patients, yet pinpointing the precise cognitive deficits causing these real-world problems has proved a challenging research question (Eslinger and Damasio, 1985; Shallice and Burgess, 1991; Tranel et al., 2007; Schneider and Koenigs, 2017). VmPFC damage disrupts tasks that broadly require future planning or sequential decision-making. This includes disruption in the multiple errand task, where patients must plan a sequence of real-world errands (Shallice and Burgess, 1991; Tranel et al., 2007), and the tower of London task, where patients plan a series of moves to a goal (Owen et al., 1990). In other simple laboratory tasks, neural correlates of planning have been observed in the orbitofrontal cortex (Rowe et al., 2001; Wilson et al., 2014; Chan et al., 2016; Schuck et al., 2016; Kaplan et al., 2017; Elliott Wimmer and Büchel, 2019; Basu et al., 2021; Costa et al., 2023). However, planning is a composite ability relying on separable cognitive components, which these tasks are not designed to tease apart. This requires planning tasks that deliver the necessary complexity to reveal subtle behavioral alterations, while still allowing researchers to discriminate between specific cognitive processes supporting planning.

Some studies have proposed that vmPFC damage causes specific impairments in imagining or simulating the future. For example, patients struggle to imagine events in the distant future (Fellows and Farah, 2005) and produce less detail about imagined future events (Bertossi et al., 2016b; 2017). On the other hand, other studies have emphasized the role of vmPFC in evaluation, particularly when choices require integrating multiple attributes of value or inhibiting irrelevant information (Camille et al., 2011; Levy and Glimcher, 2012; Bartra et al., 2013; Noonan et al., 2017; Bowren et al., 2018; Vaidya et al., 2018; Pelletier et al., 2021). New task paradigms are required to determine whether vmPFC is necessary for all computations involved in planning or whether specific aspects of planning are impaired, such as imagining the future, evaluating between states, or selecting relevant information.

The “Four-in-a-Row” task was developed to characterize the computational components of human planning in a complex state space (Ma et al., 2022; van Opheusden et al., 2023). In computational terms, planning involves using a model of the world to guide choices through simulation of possible future states of the world. This process of imagining future trajectories can be operationalized as a decision tree, where each decision is a branching point leading to alternative futures (Newell et al., 1959; Botvinick et al., 2009; Keramati et al., 2011; Otto et al., 2013; Huys et al., 2015; Sezener et al., 2019; Callaway et al., 2022; Mattar and Lengyel, 2022). Human behavior in Four-in-a-Row can be captured by a planning algorithm that separates the exploration of future states using a tree search algorithm and evaluation of the states using feature-based heuristics. By separating these distinct elements of planning, the task can characterize how far people search into the future (“depth”), their knowledge of good heuristics for evaluating states (“heuristic quality”), and their tendency to overlook relevant features (“feature drop”). Our primary aim was to determine whether vmPFC lesion patients show impairments in this complex planning task and, if so, to identify the components contributing to the deficit.

Our secondary aim was to determine whether vmPFC planning deficits depend on the complexity of the task or reflect a more general impairment in using an internal model of the world to guide choice. To investigate this ability in patients with vmPFC damage, we examined behavior in a simpler task which measures peoples’ capacity to make decisions using a model of the environment (Daw et al., 2011).

## Materials and Methods

### Participants

We studied planning behavior in three populations: lesion patients with damage to vmPFC, control lesion patients with damage outside vmPFC [lesion controls (LCs)], and healthy age-matched control participants [healthy controls (HCs)]. In both studies, we recruited lesion patients from a database of individuals who had previously visited the John Radcliffe Hospital and consented to be contacted for research studies. In Study 1, 10 vmPFC patients (age range, 46–76 years; mean age = 60.7 years; 4 females), 8 LC patients (age range, 44–67 years; mean age = 53.9 years; 5 females), and 30 HC participants (age range, 51–69 years; mean age = 58.1 years; 16 females) performed the Four-in-a-Row task. In Study 2, 30 vmPFC patients (age range, 37–78 years; mean age = 58.9 years; 17 females), 19 LC patients (age range, 32–73 years; mean age = 56.1 years; 10 females), and 20 HC participants (age range, 40–71 years; mean age = 62.0 years; 13 females) performed the Two-Step task. One LC patient was excluded from Study 2 because they failed to complete the task. Data collection for Study 2 took place 2 years prior to data collection for Study 1. With the exception of one new LC patient, all lesion patients who participated in Study 1 had previously participated in Study 2. However, since data collection for Study 1 was conducted during the Covid-19 pandemic, we were unable to test all lesion patients who had previously taken part in Study 2. All data analyses were performed after data collection was completed for both studies. The studies are presented in reverse order to highlight the main contribution of the findings. Demographic data for all groups are presented in Table 3.

For both vmPFC and LC groups, most lesions were caused by subarachnoid hemorrhage. In three cases, damage was caused by a tumor (two vmPFC and one LC), and in one LC, damage was caused by head injury. Of the 50 lesion patients who took part in the studies, 5 were taking antidepressants (3 LC, 2 on citalopram and 1 on paroxetine; 2 vmPFC, 1 on citalopram and 1 on amitriptyline), and 17 were hypertensive (12 vmPFC and 5 LC). Of the HCs, two were taking antidepressants (one on citalopram, one on amitriptyline), and three were hypertensive.

**Figure 1.**
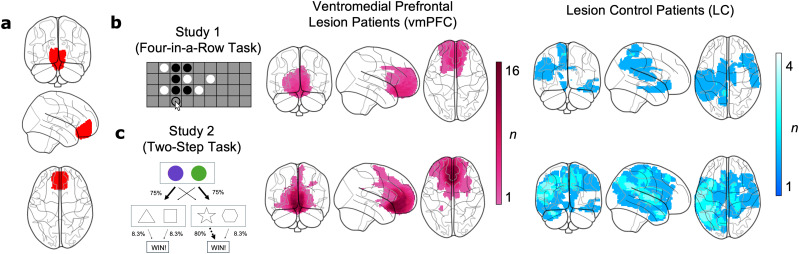
Lesion maps. ***a***, Anatomical vmPFC mask from the Harvard-Oxford Cortical Structural Atlas (RRID:SCR_001476; Kennedy et al., 1998; Makris et al., 1999) as distributed with the FMRIB Software Library (FSL; Jenkinson et al., 2012). Patients were categorized into vmPFC and LC groups on the basis of whether they had neural damage inside the mask. ***b***, Overlap of brain lesion maps for the vmPFC group (left, pink) and LC group (right, blue) who participated in Study 1 (Four-in-a-Row task). Color bar shows the number of patients with damage in each voxel. ***c***, Same as in ***b*** for Study 2 (Two-Step task).

Participants were separated into groups a priori on the basis of the location of their brain lesion damage. A neurologist (SGM) manually registered brain lesions prior to study recruitment. The Harvard-Oxford Cortical Structural Atlas (RRID:SCR_001476; Kennedy et al., 1998; Makris et al., 1999) as distributed with the Functional Magnetic Resonance Imaging of the Brain (FMRIB) Software Library (FSL; Jenkinson et al., 2012) was used to allocate participants to the vmPFC group or LC group, using the binarized mask of the frontal medial cortex for vmPFC classification (threshold > 0, Fig. 1*a*). Individuals who had damage within the mask were assigned to the vmPFC group (Study 1 vmPFC group, *n *= 10; Fig. 1*b*, left; Study 2 vmPFC group, *n *= 30; Fig. 1*c*, left), while those for whom the vmPFC was spared were assigned to the LC group (Study 1 LC group, *n *= 8; Fig. 1*b*, right; Study 2 LC group, *n *= 19, Fig. 1*c*, right). The vmPFC lesions were highly focal (median volume: 8.5 cm^3^ in Study 1 and 14.5 cm^3^ in Study 2). In Study 1 (where vmPFC damage was found to affect behavior), three vmPFC patients also had damage in the ventral striatum or dorsomedial PFC.

Ethical approval was obtained by the London Fulham Research Ethics Committee (IRAS project number, 242551; REC reference number, 18/LO/2152). All participants gave informed consent before the experiment. Participants were compensated for their time at a rate of £10 per hour.

#### Study 1 (Four-in-a-Row task)

##### Experimental methods

**Figure 2.**
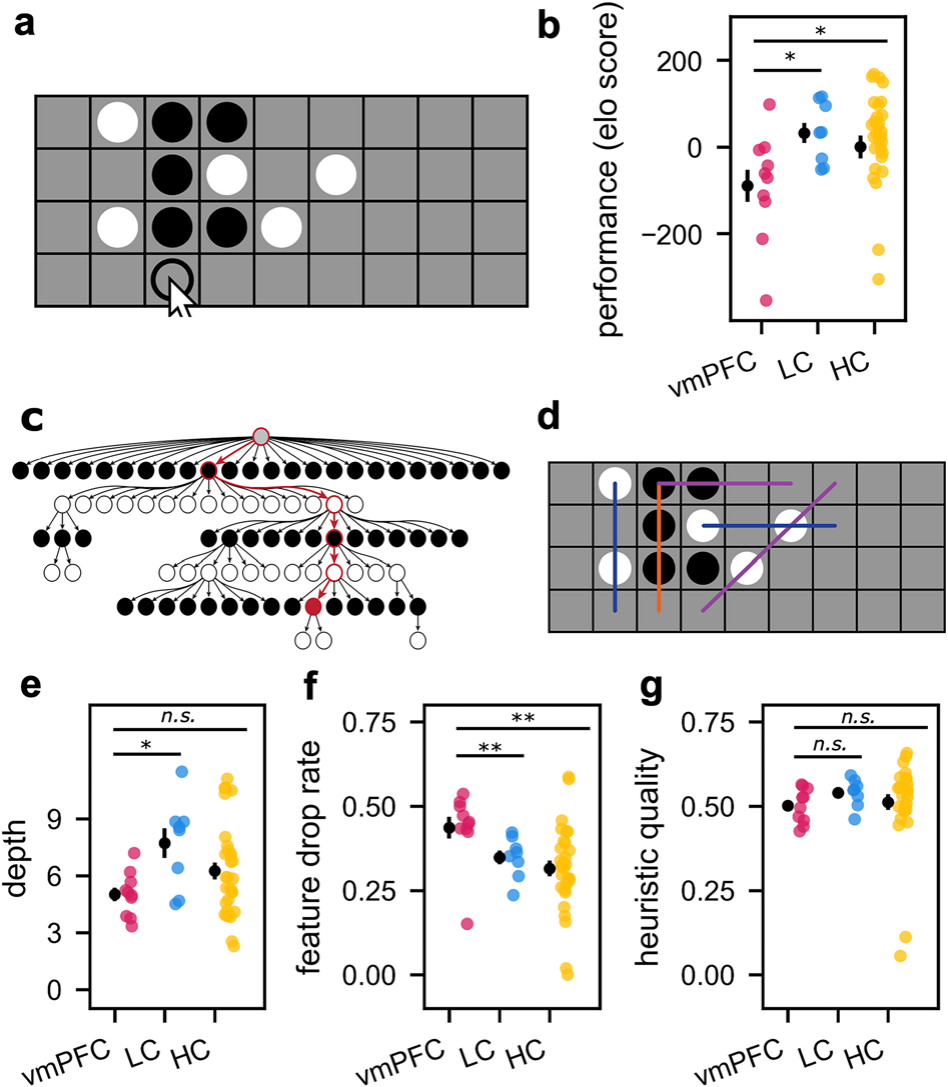
Study 1 (Four-in-a-Row task). ***a***, Depiction of the task, where participants aimed to place four pieces of their color in a row. The arrow depicts a winning move for the black player. ***b***, Elo ratings, which provide a metric of performance, or playing strength, as a function of group. Error bars show SEM, and dots show individual participant ratings. Stars show statistical significance (n.s.: *p *> 0.05; *p < 0.05; **p < 0.01) from the results of nonparametric tests comparing the vmPFC group with the control group, after establishing there was a difference across populations using a Kruskal–Wallis test. vmPFC lesion patients performed worse than both lesion controls and age-matched controls. ***c***, The computational model consists of a heuristic value function (for evaluating states) and a tree search algorithm (for simulating future moves). Depicted here is an illustration of the tree search algorithm, which constructs a partial decision tree using best-first search (Dechter and Pearl, 1985; see Materials and Methods for full description). “Depth” refers to the average length of forward search, illustrated in the example with the red trajectory. ***d***, The value function corresponds to a linear combination of heuristic features critical for playing the game. Colored lines depict example features, where purple shows connected two-in-a-row, blue shows unconnected two-in-a-row, and orange shows three-in-a-row. Within the model, “heuristic quality” refers to how closely an individual's weights for each feature match the optimal weights. “Feature drop rate” refers to the probability of overlooking a feature on the map, on any trial. ***e–g***, Summary parameters from the planning model, plotted by group. Error bars show SEM, and dots depict individual participant parameter estimates. Stars depict the result of nonparametric one-sided tests of the three hypotheses, namely, that vmPFC lesions are associated with lower depth, higher feature drop rate, and lower heuristic quality than the two control groups.

All participants played a computer-based version of “Four-in-a-Row.” In this game, two players take turns to place a single piece of their color (black or white) on an empty space in a four-by-nine grid (Fig. 2*a*). A board of this size has approximately 1.2 × 10^16^ nonterminal states (van Opheusden et al., 2023). Each player's goal is to place four pieces of their color in a line (vertical, horizontal, or diagonal) before their opponent. Our participants played against computer opponents. Each game could end in a win for the participant (the participant obtains four pieces in a row), a loss (the computer opponent obtains four pieces in a row), or a draw (the grid fills up without either player obtaining four-in-a-row). Across games, each participant alternated between playing black and white, where black always played first.

The task was programmed in JavaScript, and participants completed the game in a web browser hosted on Amazon Web Services. For the patients, the researcher remained on the telephone throughout the session to help with any technical difficulties with the task. However, all participants received identical standardized training on the web browser, which consisted of instructions, two practice games, and five comprehension questions. After training, participants completed 40 games in total. The HCs were recruited from Prolific.co.uk and received the same training and study procedure with the only difference being that the researcher was not present on the telephone during testing.

The set of AI opponents comprised 200 difficulty levels published previously (van Opheusden et al., 2023). The 200 difficulty levels were divided into five categories of playing strength (with 40 agents per category). For the two practice games, we set the initial difficulty level to 1, which is the easiest possible. After training, participants began the study by playing an opponent randomly drawn from an easy level (Category 2 i.e., Levels 40–79). Participants advanced to more challenging opponents depending on performance, as implemented using a staircase procedure. Specifically, after each game, the next opponent was chosen based on the outcome of the game: after a loss, a new opponent was drawn from the category below; after one win or a draw, a new opponent was drawn from the same category, and after two wins, a new opponent was drawn from the category above.

#### Statistical analysis

##### Task performance

We operationalized task performance as playing strength, estimated using the Elo rating system (Elo, 1978). In the Elo rating system, players are ranked based on their history of wins, losses, and draws against the same pool of opponents. In this case, we followed van Opheusden and colleagues in treating each category of computer level (five categories in total) as an individual “opponent” faced by participants (van Opheusden et al., 2023). This measure of playing strength is purely based on the history of game outcomes and neither on the quality of individual moves nor on the cognitive modeling of participant behavior.

##### Planning model

To disentangle the cognitive components of planning in Four-in-a-Row, we used the model developed and validated by van Opheusden et al. (2023). Given the size of the state space, it is impossible to plan across all possible futures in this task. For this reason, agents must search the space efficiently. To do this, the model rests on two assumptions, namely, that simple features are used to estimate the value of different moves (“heuristic value function”) and that the most promising moves are explored first during planning (“best-first search”).

The heuristic value function determines the value of each board state *V*(*s*) according to a combination of heuristic “features” (Fig. 2*d*). The algorithm posits five evaluative features: connected two-in-a-row (i.e., two consecutive pieces surrounded by empty squares such that a four-in-a-row could be formed in principle), unconnected two-in-a-row (i.e., two nonconsecutive pieces that, when combined, could form a four-in-a-row), three-in-a-row, four-in-a-row, and proximity to the board center. The model approximates the value of different moves through a weighted sum of the counts of these features across the board, regardless of location or orientation. Each feature type has a different weight *w*. In addition, features are scaled differently (with a scaling constant *C*) depending on whether their color belongs to the current “active” player or the other “passive” player during the simulated move, capturing the fact that features are more valuable if they belong to the player who is currently about to move. The final value function is as follows:

$$V(s)=w_{\mathrm{center}}V_{\mathrm{center}}+c_{\mathrm{black}}\;\underset{i\in F}{\sum }\;w_{i}f_{i}(s,\mathrm{black})\;\text{–}c_{\mathrm{white}\;}\underset{i\in F}{\sum }\;w_{i}f_{i}(s,\mathrm{white})+\varepsilon ,\;(1)$$

where F comprises the set of evaluative features listed earlier (connected two-in-a-row, unconnected two-in-a-row, three-in-a-row, four-in-a-row), $c_{\mathrm{black}}=C$ and $c_{\mathrm{white}}=1$ whenever black is to move in state *s*, and $c_{\mathrm{black}}=1$ and $c_{\mathrm{white}}=C$ whenever white is to move in state *s*. The last term ε adds Gaussian noise with mean zero and unit variance.

Guided by the value function, the tree search algorithm constructs a partial decision tree using best-first search (Fig. 2*c*; Dechter and Pearl, 1985). On each iteration, the value function determines which position to explore, resulting from the sequence if both players choose their highest-value moves in the current tree. All legal moves from the selected position are evaluated, and values are backpropagated to predecessor nodes up to the root of the tree using the minimax rule. Moves that are lower than the best move minus a threshold (*θ*) are pruned. This reflects the fact that people cannot do an exhaustive search over the state space and aligns with empirical evidence that people “prune” branches with initial low values (Huys et al., 2012). Finally, at the end of each iteration, there is a probability of the search being terminated with a stopping probability parameter.

In addition to the parameters related to the value function and tree search, there are two additional parameters related to sources of noise. The “feature drop” parameter accounts for limitations of selective attention. Specifically, it is the probability of missing a feature on a particular trial (a particular feature is dropped from *V*(*s*) at all points in the tree). Finally, the lapse rate is the probability of choosing a random move. All parameters are summarized in Table 1.

**Table 1.** Parameters from the Four-in-a-Row model

Parameter	Symbol	Description
Center weight	$w_{\mathrm{center}}$	Weight of the center feature, which prioritizes squares near the center of the board
Connected two-in-a-row weight	$w_{2\mathrm{conn}}$	Weight of the feature that recognizes adjacent pieces with enough surrounding space to create four-in-a-row
Unconnected two-in-a-row weight	$w_{2\mathrm{unc}}$	Weight of the feature that recognizes nonadjacent pieces with enough surrounding space to create four-in-a-row
Three-in-a-row weight	$w_{3\mathrm{inarow}}$	Weight of the feature that recognizes three-in-a-row with the remaining square unoccupied
Four-in-a-row weight	$w_{4\mathrm{inarow}}$	Weight of the feature that recognizes four-in-a-row when it is present (terminal states only). This weight is almost always maximal
Active–passive scaling factor	C	The multiplicative constant applied to the weight of the player whose move it is, capturing the intuition that having two-in-a-row or three-in-a-row is more valuable during the participant's turn since they can exploit the pattern
Pruning threshold	θ	Value threshold below which moves are excluded from consideration entirely, even if other candidate moves end up having lower value after planning
Stopping threshold	γ	The probability of terminating the search after each iteration in the planning algorithm. Intuitively, the total number of iterations scales as 1/ γ and the depth scales as log(γ), though these relationships are only approximate
Feature drop rate	δ	The probability of a participant overlooking a given feature instance in a given state. Features are dropped independently across states, participants, and feature instances
Lapse rate	λ	The probability that the model bypasses the entire search algorithm and moves randomly. This parameter is mostly present for numerical stability

Description of all free parameters in the Four-in-a-Row planning model, fitted to each participant separately. Summary metrics were derived from these parameters and are presented in Table 2. Analyses are performed on summary metrics rather than parameters.

To fit the model to individual participants, we optimized the log likelihoods of our models using Bayesian adaptive direct search (BADS), with fivefold cross-validation (Acerbi and Ma, 2017). For each participant, we then converted the set of 10 parameters (five feature weights, the scaling factor *C*, the pruning threshold, stopping probability, feature drop rate, and lapse rate) to 3 final summary parameters (depth, heuristic quality, and feature drop rate), which have better reliability and test–retest stability than the basic model parameters (van Opheusden et al., 2023). These summary metrics are described in Table 2, and are functions of the model parameters only. Following the original methods of van Opheusden et al. (2023), they are calculated by simulating the model's behavior on a fixed set of 5,482 probe states. The probe states consist of real states in human versus human tournaments previously selected and validated in van Opheusden et al. (2023). We repeated this simulation over all states 10 times, to minimize variability in noise. The three summary parameters are described below:

1. Planning depth: This parameter captures the depth of the decision tree and can be thought of as the number of moves a player plans into the future. To calculate decision tree depth for each participant, we use their individual parameters to generatively run the model forward on the probe states. For each simulated move, we measure the length of the principal variation, that is, the sequence of highest-value moves for both players, until a leaf node in the tree is reached. Specifically, for each board position, we generated 10 simulations using the fitted parameters. In each simulation, we stored the depth of the sequence of moves considered best. Next, we averaged this across simulations and board positions. An individual's planning depth is defined as the average length of the principal variation across all probe states and across all repetitions.
2. Heuristic quality: Heuristic quality reflects the correlation between the participant's subjective value (given the state and feature weights) and the objective value from the optimal weights. The subjective value is calculated for each state using a participant's weighted combination of features. The optimal state values were calculated by running the model with no noise and no pruning until convergence on the state value. Heuristic quality is the correlation between the subjective state value (the participant's weighted combination of features) and the objective optimal value. Importantly, the heuristic quality depends on the feature weights in the model but does not rely on the parameters of the tree search algorithm, which affects planning depth.
3. Feature drop rate: This parameter directly corresponds to an estimated parameter in the model. This parameter estimate reflects the probability that the agent overlooks a random feature and temporarily drops it from the value function. When a feature is “dropped,” its weight is temporarily set to zero during a particular move. Feature drop rate therefore measures oversights of relevant features during gameplay.

**Table 2.** Summary metrics for Four-in-a-Row

Metric	Description
Planning depth	Average depth of the principal variation in probe positions. Intuitively, the depth to which the model with inferred parameters makes a plan for the future.
Feature drop rate	Probability of overlooking a feature. This metric is simply the feature drop rate parameter itself
Heuristic quality	Correlation between value function estimated using the feature-based function (Eq. 1) and the objective game-theoretic value, calculated across probe states

For each participant, the model parameters in Table 1 are used to generate three final summary parameters: depth, heuristic quality, and feature drop rate. These summary parameters capture the main behaviors of interest and have better reliability and test–retest stability than the basic model parameters (van Opheusden et al., 2023).

Model fitting was performed on the NYU high-performance cluster (Intel Xeon E5-2690v2 CPUs 3.0 GHz) with a parallel implementation of inverse binomial sampling, which uses 20 cores.

##### Group comparisons

Across all group comparisons, we used nonparametric tests because all variables violated assumptions of normality. First, we established whether the groups differed in performance using a Kruskal–Wallis test to determine if the Elo rating differed as a function of lesion group (vmPFC, LC, or HC). We followed this test with two-sided Mann–Whitney *U* tests. The critical test was whether individuals with vmPFC lesions differed from other individuals with lesion damage (vmPFC patients vs LC) and, following this, from healthy age-matched controls (vmPFC patients vs HC).

As an additional control, we verified that performance was truly related to the location of damage rather than the size of the lesion. To do this, we predicted Elo ratings using the volume of brain damage within the vmPFC, while controlling for the total volume of brain damage:

$$\mathrm{Elo}\;∼\alpha \;+\beta_{0}V_{\mathrm{vmPFC}}+\beta_{1}V_{\mathrm{total}}\;+\varepsilon ,\;(2)$$

where $V_{\mathrm{vmPFC}}$ refers to the volume of brain damage within the vmPFC while $V_{\mathrm{total}}$ refers to the total volume of brain damage (where volume was quantified in voxels). Once we established that vmPFC lesion patients had a performance deficit, we used a one-sided Mann–Whitney test to test three hypotheses, namely, that vmPFC lesion patients planned less deeply into the future (depth), were more likely to miss valuable features (feature drop rate), or demonstrated worse heuristic value estimates (heuristic quality). Again, we started with the critical test to determine whether there was an effect of vmPFC lesion within the lesion population (vmPFC patients vs LC), following up with a comparison against healthy age-matched controls (vmPFC patients vs HC). We addressed the potential confounding impact of age and education on performance by conducting additional control analyses. Within the HC group, we used multiple linear regression to estimate the impact of age and education on our four metrics (Elo score, depth, feature drop rate, and heuristic quality). The regression coefficients were then used to predict metrics across all groups, and residualized scores were calculated by subtracting the predictions from the observed values. We then repeated the statistical analyses described above on the residualized scores, to control for the contribution of age and education.

#### Study 2 (Two-Step task)

##### Experimental methods

**Figure 3.**
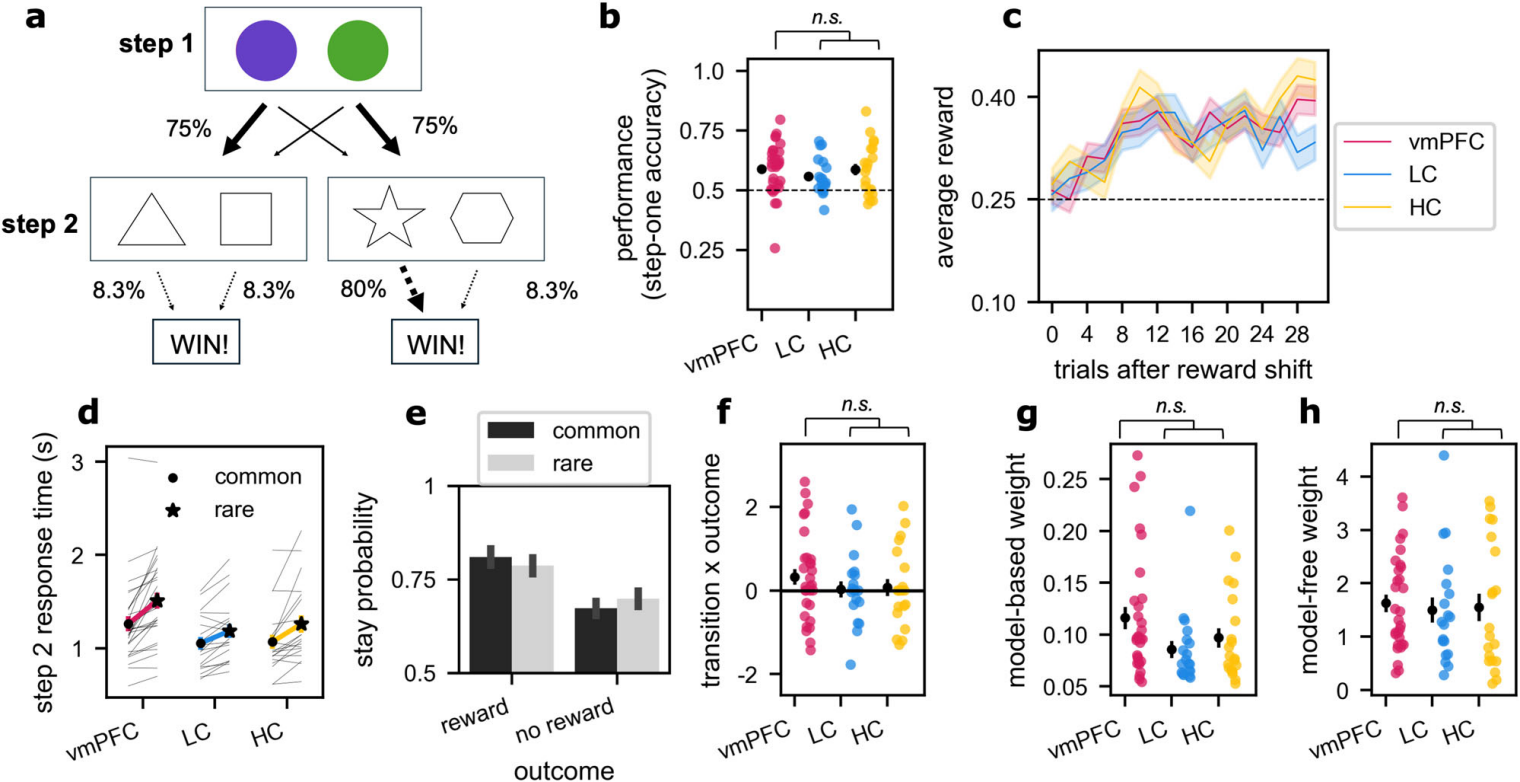
Study 2 (Two-Step task). ***a***, Depiction of the task, where participants made two sequential decisions between colors (Step 1) followed by shapes (Step 2), with the aim of maximizing wins. The arrows between Step 1 and Step 2 illustrate the common transitions (bold arrow, 75% probability) and the rare transitions (narrow arrow, 25% probability). Arrows between Step 2 and the reward outcome illustrate the probability of winning after selection of each shape. At any point in time, a particular shape (in this example, the star) was associated with a high probability of winning reward. The high-reward option shifted to a different shape every 32 trials. ***b***, Performance plotted by group, quantified as the proportion of correct choices at Step 1 which corresponds to choosing the first step option that commonly led to the rewarded shape. Error bars depict SEM, dots show individual data points, and the line depicts chance performance. Significance bars show the result of the ANOVA test for effect of lesion group (n.s. corresponds to *p *> 0.05). ***c***, Average proportion of rewarded trials plotted by the trial number following a shift in reward contingency. The rewarded shape changed every 32 trials. Color depicts groups, and confidence intervals depict SEM. ***d***, Step 2 choice response times plotted for the three groups (color), split by whether the transition experienced was common or rare (circle shows common, and star shows rare). Colored lines show group averages, and pale lines show response times for individual participants. Within all groups, participants slow down following a rare versus common transition, showing sensitivity to the general transition probabilities in the task. ***e***, Analysis of stay probabilities: The probability of repeating the same choice is plotted as a function of the previous outcome (reward or no reward) and the previous transition experienced (common or rare). ***f***, Modulation of stay behavior as a function of previous outcome and transition. The outcome–transition interaction *β* weights are plotted by lesion group, where higher *β* values indicate more “model-based” modulation of behavior. Error bars depict SEM, and dots show individual data points. Significance bars show the result of the Kruskal–Wallis test for effect of lesion group. ***g***, Model-based weights from the reinforcement learning model capturing the contribution of model-based strategy. Annotations show the result of nonparametric tests for differences between populations. Model-based weights are plotted by group, whereas error bars depict SEM. Dots show individual data points. Significance bars show the result of the Kruskal–Wallis test for effect of lesion group. ***h***, Same as in ***g*** for model-free weights.

All participants completed a variant of the Two-Step task (Daw et al., 2011), designed to measure habitual versus goal-directed decision-making. Data collection took place in person, at the John Radcliffe Hospital in Oxford. The task involved making repeated two-stage decisions in order to earn rewards (Fig. 3*a*). On each trial, participants first chose between two colors and then between two shapes. Of crucial significance to the task, each color in Step 1 led to a specific pair of shapes in Step 2 with a 75% probability (“common transition”) but led to the opposite pair of shapes in 25% of trials (“rare transition”). Of the four possible shapes that could be offered in Step 2, only one shape had a high probability of reward at any point in time. This required participants to think strategically about which choice in Step 1 was most likely to lead them to the set of offers that included the high-reward option.

A learner who uses a model-free strategy will be more likely to repeat their Step 1 choice on the next trial after being rewarded at Step 2, regardless of whether the previous transition between steps was common or rare. However, a decision-maker who uses a model of the task structure will be sensitive to the relationship between the steps. For example, they should repeat their Step 1 choice after being rewarded on a common transition but switch choices when rewarded on a rare transition, which informs them that the opposite Step 1 choice is rewarding.

To facilitate learning in the patient population, the reward probabilities for the Step 2 choices were stationary for long periods with abrupt shifts in reward (as in Akam et al., 2015; Castro-Rodrigues et al., 2022; Doody et al., 2022; Blanco-Pozo et al., 2024), rather than drifting continuously. Specifically, at any point in time, one arm would be associated with a high reward probability (80% chance of payout) while each of the other three arms would be associated with low reward probabilities (8.3% chance of payout). The high-reward option was associated with the same arm for a period of 32 trials, before switching to a different arm (unannounced to the participant). The entire study consisted of 288 trials (nine blocks of 32 trials). Participants received standardized instructions from the experimenter in person. The task was coded in MATLAB.

#### Statistical analysis

##### Performance and simple behavioral analyses

We operationalized performance as the proportion of correct choices for Step 1, i.e., choices that, if the common transition occurred, would lead to the rewarding shape in Step 2. Given the probabilistic reward structure of the task, this metric of performance is less noisy than the overall reward. We examined Step 1 choices rather than Step 2 choices because only Step 1 choices can capture planning across the two steps. We used nonparametric Kruskal–Wallis tests to determine whether there was a difference between groups.

Next, we quantified the extent to which participants were sensitive to the transition structure of the environment through response times. Participants using a model of the environment may slow down more when making their Step 2 choice after surprising rare transitions compared with predicted common transitions (Nussenbaum et al., 2020). For each participant, the average Step 2 response times following a rare transition and following a common transition were computed. Paired *t* tests were used to determine whether response times differed as a function of transition.

Second, we analyzed stay probability to assess model-free versus model-based behavioral strategies (Daw et al., 2011; Otto et al., 2013; Friedel et al., 2014; Eppinger et al., 2013; Worbe et al., 2016; Lockwood et al., 2020; Castro-Rodrigues et al., 2022). We examined the probability that people would repeat their Step 1 choice as a function of the previous outcome (reward vs no reward) and previous transition (common vs rare) experienced. When deciding whether to repeat their choice, model-based agents will not only take into account whether they were rewarded but will modulate this by whether the reward outcome followed a common versus rare transition sequence. We quantified this in a logistic regression model, where the previous outcome, transition, and transition–outcome interaction were all used as predictors of staying on the subsequent Step 1 choice. In addition, we included a binary control regressor capturing the tendency to repeat correct Step 1 choices (i.e., whether the Step 1 choice on the previous trial commonly leads to the high-rewarded state). This correct predictor was included following Akam et al. (2015), who showed that analyses of stay probabilities in the Two-Step task can give rise to inflated metrics of model-based strategies unless this control regressor is included (Akam et al., 2015). Following previous studies, we used the weights corresponding to the transition–outcome interaction as a marker of model-based reasoning (Daw et al., 2011; Akam et al., 2015).

##### Reinforcement learning model

We modeled choices using a reinforcement learning model with separate components capturing model-based and model-free learning (Daw et al., 2011). The task involves three states, with only one Step 1 state and two possible Step 2 states. In the following notation, $s_{1,t}$ corresponds to the Step 1 state taken at trial *t* (which is always the same), while $s_{2,t\;}\;$corresponds to the Step 2 state (dependent on the first choice and transition). In each state, there are two available actions ( $a_{A}$ or $a_{B}$).

##### Model-free algorithm

The model-free algorithm updates the value of state–action pairs according to a SARSA (λ) temporal-difference reinforcement learner (Daw et al., 2011; Rummery and Niranjan, 1994). At each step *i* of the Two-Step trial *t*, the value for the chosen action $\left(a_{i,t}\right)$ is updated as follows:

$$Q_{\mathrm{mf}}(s_{i,t},a_{i,t})\leftarrow Q_{\mathrm{mf}}(s_{i,t},a_{i,t})+\;\alpha \delta_{i,t},\;(3)$$

where α is a learning rate parameter and the reward prediction error (RPE; $\delta_{i,t}$) corresponds to the following:

$$\delta_{i,t}=\;r_{i,t}+\;Q_{\mathrm{mf}}(s_{i+1,t},a_{i+1,t})-\;Q_{\mathrm{mf}}(s_{i,t},a_{i,t}),\;(4)$$

The reward following the Step 1 choice $\left(r_{1,t}\right)$ is always 0, while the reward following the Step 2 choice $\left(r_{2,t}\right)$ can be 1 or 0. Note that the prediction error is driven by different sources of information after the first versus second stage choices. At the Step 1 choice, the reward is never received, so the update is driven by the Step 2 value, $Q_{\mathrm{mf}}(s_{2,t},a_{2,t})$. At the Step 2 choice, the update is driven entirely by the reward received, $r_{2,t}$ (while the value of the subsequent state is set to zero because the trial ends after two steps). Finally, at the end of the trial, the value of the Step 1 choice is also updated with an eligibility trace. In other words, the RPE from the final choice is used to update the Step 1 choice, multiplied by an eligibility parameter (*λ*; Sutton and Barto, 2018):

$$Q_{\mathrm{mf}}(s_{1,t},a_{1,t})=Q_{\mathrm{mf}}(s_{1,t},a_{1,t})+\;\lambda \alpha \delta_{2,t},\;(5)$$

##### Model-based algorithm

The model-based algorithm updates its values for Step 1 using a model of the task structure—that is, the probabilities associated with transitioning between steps. For example, if the state in Step 2 was unlikely to occur after the Step 1 choice (rare transition of 25%), the algorithm correspondingly updates the value of the action in Step 1 that most commonly reaches the rewarded state. Below, $s_{A}$ and $\;s_{B}\;$ denote the two possible second states. The values of the Step 1 actions $\left(a_{j}\right)$ are computed according to the Bellman equation:

$$Q_{\mathrm{mb}}(s_{1},a_{j})=P(s_{A}|s_{1},a_{j})\underset{a\in a_{A},a_{B}\;}{\max}\;Q_{\mathrm{mf}}(s_{A},a)+\;P(s_{B}|s_{1},a_{j})\underset{a\in a_{A},a_{B}\;}{\max}\;Q_{\mathrm{mf}}(s_{B},a),\;(6)$$

This is recomputed at every trial from the current estimates of value. In Step 2, model-based learning is equivalent to model-free learning, since the second step value purely reflects an estimate of the immediate reward (Daw et al., 2011).

##### Choice algorithm

The influence of model-based versus model-free strategies can be quantified in the choices in Step 1. The probability of choosing each Step 1 action is determined by a combination of model-based value, model-free value, and a repetition bias. We fit an adapted version of Daw's original model that has previously been used to investigate individual differences in the Two-Step task (Decker et al., 2016; Potter et al., 2017; Nussenbaum et al., 2020). Specifically, this model contains separate softmax temperature parameters associated with the influence of the model-based value $\left(\beta_{\mathrm{mb}}\right)$ and the model-free value $\left(\beta_{\mathrm{mf}}\right)$, alongside a parameter capturing a bias to repeat the Step 1 choice from the previous trial (p). The probability of choosing each possible action $a_{i}$ in Step 1 is as follows:

$$P(a_{i}|s_{1,t})=\frac{\exp(\beta_{\mathrm{mf}}\;*\;Q_{\mathrm{mf}}(s_{1,t},a_{i})+\;\beta_{\mathrm{mb}}\;*\;Q_{\mathrm{mb}}(s_{1,t},a_{i})+p\;*\;\mathrm{rep}(a_{i}))}{\sum_{a^{\prime }}\;\exp(\beta_{\mathrm{mf}}\;*\;Q_{\mathrm{mf}}(s_{1,t},a^{\prime })+\;\beta_{\mathrm{mb}}\;*\;Q_{\mathrm{mb}}(s_{1,t},a^{\prime })+p\;*\;\mathrm{rep}(a^{\prime }))},\;(7)$$

In Step 2, the model-free value is used to predict choice with a separate softmax temperature:

$$P(a_{i}|s_{2,t})=\frac{\exp(\beta_{\mathrm{Step}\;2}\;*\;Q_{\mathrm{mf}}(s_{2,t},a_{i})}{\sum_{a^{\prime }}\;\exp(\beta_{\mathrm{Step}\;2}\;*\;Q_{\mathrm{mf}}(s_{2,t},a^{\prime })},\;(8)$$

The final model had six free parameters, namely, a Step 1 weight for model-free value $\left(\beta_{\mathrm{mf}}\right)$, a Step 1 weight for model-based value $\left(\beta_{\mathrm{mb}}\right)$, a Step 1 weight for persistence (repeating the previous choice; p), a Step 2 softmax temperature for model-free value $\left(\beta_{\mathrm{Step}\;2}\right)$, learning rate (α), and eligibility parameter (λ).

##### Model fitting and validation

We used a Bayesian hierarchical modeling framework to fit the reinforcement learning models to behavior, allowing us to pool data across participants to improve individual parameter estimates. We coded the models in the Stan modeling language (Carpenter et al., 2017), fitting each dataset using the CmdStanPy interface. To aid model fitting in stan, we used reparameterization to sample parameters from centered standard normal distributions (which facilitate gradient calculations in stan), which were then transformed into the appropriate prior distributions. Group-level variances were defined as lognormal distributions to ensure only positive values. For all parameters with the exception of *p* (for which the appropriate prior distribution is a centered normal distribution), parameter transformations were used to enforce constraints and impose uniform prior distributions across the appropriate ranges. Parameters were transformed using an approximation of the phi function (i.e., normal cumulative density function), which leads to a uniform prior over the constrained range when applying the cumulative density function to a normal distribution. We constrained α and λ to have a uniform prior on (0,1) and constrained $\beta_{\mathrm{mb}}$, $\beta_{\mathrm{mf}}$, and $\beta_{\mathrm{Step}\;2}$ to have a uniform prior on (0,10). The individual-level parameters for the *i*th participant (*p^i^*, $\alpha^{i}$, $\lambda^{i}$, $\beta_{\mathrm{mb}}^{i}$, $\beta_{\mathrm{mf}}^{i}$, and $\beta_{\mathrm{Step}\;2}^{i}$) were given a normal distribution with the mean as the prior on group mean and variance as the prior on group variance. The individual-level parameters were then also transformed using the phi function to enforce constraints. Datasets were fit with four chains, using 1,000 samples per chain (warmup, 500). *R*-hat values ≤ 1.1 indicated convergence across all parameters. Following previous studies (Decker et al., 2016; Potter et al., 2017), we did not include the first nine choice trials in the analysis.

Since previous studies have shown that model-free behavior can be mistaken for model-based behavior in environments with stationary probabilities (Akam et al., 2015), it was important to validate the model to determine that model-based behavior could still be recovered in the task variant used in this study. Behavior was simulated for the 70 participant schedules (transitions and reward probabilities), repeated 10 times each. On each iteration, the six parameters were sampled from normal distributions with means and standard deviations reported in previous studies (Decker et al., 2016). The simulated data were then fit using the same procedure described above, used for the empirical data. Parameter recoverability was high across all six parameters (all Pearson's *R *> 0.81, all *p *< 0.001).

##### Group comparisons

Following the same analysis procedure as Study 1, we began by investigating differences in performance between the three groups (vmPFC, LC, and HC), defined for this task as accuracy for the Step 1 choice. ANOVA was used since this metric did not violate assumptions of normality.

We then investigated group differences in model-based planning. Three metrics were used to probe model-based planning. First, we used analysis of stay probabilities to quantify whether participants took into account the transition structure of the task. Specifically, this is the interaction between whether a choice was rewarded and whether the outcome followed a common or rare transition (transition–outcome interaction on the probability of staying). Second, we quantified sensitivity to the transition structure by analyzing response time differences for making a Step 2 decision following a rare versus common transition after Step 1. This was computed as an individual's difference in their average Step 2 response time following a rare versus common transition, where a smaller difference could indicate lower sensitivity to the task model. Finally, we formally quantified model-based planning weights using the reinforcement learning model described above. To test for differences as a result of lesion groups, we used ANOVA if assumptions of normality were not violated and nonparametric Kruskal–Wallis tests otherwise.

## Results

### Demographics and neuropsychological assessments

Demographic information and statistical comparisons for controls and patient groups are presented in Table 3. With the exception of one LC, all lesion patients who participated in Study 2 also participated in Study 1. As independently assessed for the groups in both studies, there was no difference between any pairs of groups in terms of gender (Fisher's exact test for comparing proportions, all *p *> 0.523), age (independent *t* tests, uncorrected, all *p *> 0.065), or apathy [Apathy Motivation Index (AMI), Ang et al., 2017; independent *t* tests, uncorrected, all *p *> 0.130; excluding comparisons with the HC group in Study 1, for which we were not able to collect AMI data]. For both tasks, there was also no difference in education (education level, independent *t* tests, uncorrected, both *p *> 0.690) or depression (Beck Depression Inventory, Beck et al., 1961; independent *t* tests, uncorrected, both *p *> 0.423) between the two lesion patient groups. However, the vmPFC and LC groups had lower levels of education than the HC groups. Controlling for education and age did not affect our findings, as detailed below.

**Table 3.** Demographic information for controls and lesion patients

	Age (years)	Gender (F/M)	Education (level)^a^	Apathy (AMI)	Depression (BDI)
Two-Step					
Healthy controls (*n *= 20)	62.0 ± 9.0	13/7	4.1 ± 1.0	1.1 ± 0.6	5.2 ± 4.9
vmPFC (*n *= 30)	58.9 ± 10.8	17/13	3.0 ± 1.1^b^	1.4 ± 0.6^b^	10.4 ± 7.6^b^
Lesion controls (*n *= 19)	56.1 ± 9.9	10/9	3.1 ± 1.2	1.4 ± 0.6	12.6 ± 10.6
vmPFC–LC	0.383	1.000^a^	0.690	0.964	0.423
vmPFC–HC	0.299	0.768^a^	0.001*	0.130	0.011*
LC–HC	0.065	0.523^a^	0.009*	0.195	0.010*
Four-in-a-Row					
Healthy controls (*n *= 30)	58.1 ± 4.8	16/14	4.1 ± 1.7^d^	–	–
vmPFC (*n *= 10)	60.7 ± 9.8	4/6	2.9 ± 1.1^c^	1.5 ± 0.7^c^	11.0 ± 9.8^c^
Lesion controls (*n *= 8)	53.9 ± 6.8	5/3	2.9 ± 1.1^c^	1.5 ± 0.4^c^	13.6 ± 9.0^c^
vmPFC–LC	0.133	0.637^a^	0.958	0.913	0.622
vmPFC–HC	0.280	0.716^a^	0.079	–	–
LC–HC	0.060	0.709^a^	0.107	–	–

Values in the top rows correspond to means ± SDs. Values in the bottom rows correspond to *p*-values for independent *t* tests for group comparisons, uncorrected (unless otherwise indicated). Notably vmPFC lesion patients have significantly lower education levels than other groups. Given this possible confound, we include a control analysis showing our results are not driven by differences in education (or age). F, female; M, male.

a *p*-values for Fisher's exact test for comparing two proportions.

b Data missing for two patients.

c Data missing for one patient.

d Value based on data for 17/30 Four-in-a-Row HCs. Data about education for this group were collected subsequent to main data collection and are therefore incomplete.

* Statistically significant difference at *p *< 0.05.

We also compared the performance of LC patients and vmPFC patients across a range of neuropsychological assessments, summarized in Table 4. The two patient populations were matched across all assessments including the Corsi block-tapping task for measuring working memory (Milner, 1971), Wechsler Test of Adult Reading (Weschler, 2001), Raven's Progressive Matrices (Raven, 1938/1956), Trail Making Test (Reitan, 1958), and Addenbrooke's Cognitive Examination (Mioshi et al., 2006; all *p *> 0.294).

**Table 4.** Performance on neuropsychological assessments for vmPFC and control lesion patients

Neuropsychological assessment	vmPFC (*n *= 30)	LC (*n *= 19)^a^	*t* ^c^	df	*p*
	mean ± SD	mean ± SD			
Corsi block-tapping spatial span	5.4 ± 0.5	5.5 ± 0.5	−0.73	47	0.467
Wechsler Test of Adult Reading	37.1 ± 9.6^b^	38.1 ± 9.5	−0.34	46	0.739
Raw/standard	99.0 ± 17.5^b^	100.7 ± 17.1	−0.32	46	0.750
Raven's Progressive Matrices	3.4 ± 2.2	3.7 ± 2.5^b^	−0.38	46	0.709
Trail Making A/B (seconds)	28.1 ± 10.5	27.8 ± 13.2	0.07	47	0.941
	76.4 ± 48.7	80.9 ± 58.6	−0.28	47	0.778
ACE (total)	90.6 ± 7.6	91.6 ± 7.6	−0.44	47	0.659
ACE attention (/18)	16.3 ± 2.1	16.5 ± 1.8	−0.29	47	0.774
ACE memory (/26)	22.6 ± 3.9	22.6 ± 4.7	−0.01	47	0.992
ACE fluency (/14)	11.4 ± 2.1	11.8 ± 4.0	−0.53	47	0.598
ACE language (/26)	25.1 ± 1.0	25.4 ± 0.9	−1.06	47	0.294
ACE visuospatial (/16)	15.3 ± 0.9	15.2 ± 1.1	0.187	47	0.852

Values correspond to means ± SDs.

a Neuropsychological assessment data are missing from one lesion control patient who participated in the Four-in-a-Row study only. Scores for all other patients across both studies are included unless otherwise indicated by ^b^.

b Data from one patient missing.

c Two-tailed independent *t* test between group scores.

### vmPFC damage impairs complex planning in Four-in-a-Row

vmPFC lesion patients were worse at playing Four-in-a-Row compared with both control lesion patients and age-matched healthy controls (Fig. 2*b*). To quantify playing strength, we used the Bayeselo algorithm (https://www.remi-coulom.fr/Bayesian-Elo/), originally developed for rating chess players, which has previously been used to rate performance in Four-in-a-Row (van Opheusden et al., 2023). A Kruskal–Wallis test indicated that there was a difference in Elo ratings between he three groups (*H*_(2)_ = 7.20, *p *= 0.027). Lesion patients with vmPFC damage had lower Elo ratings compared with LCs (median vmPFC Elo rating = −66.0, median LC Elo rating = 33.5; two-sided Mann–Whitney; *n*_1 _= 10, *n*_2 _= 8, *U *= 14, *p *= 0.021) and also compared with HCs (median HC Elo rating = 27.5; two-sided Mann–Whitney; *n*_1 _= 10, *n*_2 _= 30, *U *= 71, *p *= 0.014).

To eliminate the possibility that our results were driven by differences in lesion size rather than location, we controlled for total lesion volume in a regression analysis. Within the patient population, we found that lower Elo ratings were predicted by larger vmPFC lesions (*β* = −0.08, *p *= 0.033) but not significantly by larger lesions in general (*β* = 0.00, *p *= 0.664). These findings suggest that damage to the vmPFC impairs performance in this complex planning task.

### Planning deficits following vmPFC lesions are linked to attentional oversights and lower depth of search

How could vmPFC lesions affect planning? We fit behavior in the Four-in-a-Row task using a computational process model, allowing us to investigate three separate cognitive components of complex planning. vmPFC patients could be worse at Four-in-a-Row because they plan less far into the future (lower “depth”), struggle to evaluate moves using heuristics (lower “heuristic quality”), or overlook relevant features during planning (higher “feature drop rate”).

The “feature drop” parameter captures the probability of overlooking relevant information on the board when planning a move. We found the vmPFC group was more likely to miss important features on the board compared with both LCs (Fig. 2*f*; one-sided Mann–Whitney; *n*_1 _= 10, *n*_2 _= 8, *U *= 72, *p *= 0.002) and HCs (one-sided Mann–Whitney; *n*_1 _= 10, *n*_2 _= 30, *U *= 246, *p *= 0.001).

The “depth” parameter captures how far into the future participants were planning. We found the vmPFC group planned to a lower depth than LCs (Fig. 2*e*; one-sided Mann–Whitney; *n*_1 _= 10, *n*_2 _= 8, *U *= 15, *p *= 0.013). This result did not survive comparison with HCs (one-sided Mann–Whitney; *n*_1 _= 10, *n*_2 _= 30, *U *= 103, *p *= 0.073), although depth was lower for vmPFC patients when compared with both control groups pooled (one-sided Mann–Whitney; *n*_1 _= 10, *n*_2 _= 38, *U *= 118, *p *= 0.035).

The “heuristic quality” parameter captures the difference between a participant's use of appropriate heuristics to evaluate moves and how an optimal player would use these heuristics (e.g., assigning a high value to a three-in-a-row feature of one’s own color). Damage to vmPFC did not significantly impair the quality of the heuristics used to evaluate moves compared with either LCs or HCs (Fig. 2*g*; one-sided Mann–Whitney; LCs: *n*_1 _= 10, *n*_2 _= 8, *U *= 23, *p *= 0.072; HCs: *n*_1 _= 10, *n*_2 _= 30, *U *= 104, *p *= 0.078).

The relationship between vmPFC damage and planning performance is unlikely to be driven by differences in age or education. To address these potential confounds, we first estimated the influence of age and education on our behavioral metrics in the healthy control group. Using the resulting regression coefficients, we removed the estimated contribution of age and education to behavior across all groups by residualizing the behavioral metrics. Repeating the analyses on these residualized scores confirmed our main findings: Elo scores were lower in the vmPFC group compared with both control groups (two-sided Mann–Whitney; LC: *n*_1 _= 10, *n*_2 _= 8, *U *= 5, *p *= 0.0008; HC: *n*_1 _= 10, *n*_2 _= 30, *U *= 59, *p *= 0.004), feature drop rate was higher in the vmPFC group compared with both control groups (one-sided Mann–Whitney; vmPFC > LC: *n*_1 _= 10, *n*_2 _= 8, *U *= 67, *p *= 0.008; vmPFC > HC: *n*_1 _= 10, *n*_2 _= 30, *U *= 225, *p *= 0.010), and depth was lower in the vmPFC group compared with the LC group but not the HC group (one-sided Mann–Whitney; vmPFC < LC: *n*_1 _= 10, *n*_2 _= 8, *U *= 7, *p *= 0.001; vmPFC < HC: *n*_1 _= 10, *n*_2 _= 30, *U *= 113, *p *= 0.127). While we addressed potential confounds of age and education by accounting for their contribution through linear regression, we acknowledge that assuming a linear relationship between these variables may not fully capture their effects in our small sample.

### Two-Step task showed no evidence of model-based planning in patients or controls

We found that vmPFC patients were impaired at planning in the complex Four-in-a-Row task and had a tendency to overlook relevant information and to search less deeply into the future. Next, we asked whether behavioral differences following vmPFC lesions could be detected in a simpler task which also probes the use of an internal model to make choices—a prerequisite for more complex planning. We adopted the “Two-Step” task (Daw et al., 2011), originally developed to measure decisions reflecting the use of a model of the environment (“model-based”) from more basic decision strategies (“model-free”). In this simpler planning setting, decisions affect outcomes at most two steps into the future and involve choices between binary options with only a single attribute.

All three groups performed above chance in the task, but showed no difference as a function of lesion location. In each group, participants picked the correct color at Step 1 more frequently than chance (Fig. 3*b*; one-sample two-sided *t* test comparing the mean proportion of correct Step 1 choices against 0.5; vmPFC: *t*_(29)_ = 4.44, *p *= 0.0001; LCs: *t*_(18)_ = 3.23, *p *= 0.005; HCs: *t*_(19)_ = 3.49, *p *= 0.002), showing a basic understanding of the reward structure. However, unlike in the Four-in-a-Row task, lesion damage did not significantly affect performance (ANOVA for effect of lesion group on Step 1 choice accuracy; *F*_(2,66)_ = 0.60, *p *= 0.553).

While we found no evidence that vmPFC damage affected performance, lesions may affect the type of strategy used in the Two-Step task. On each trial, receiving reward depends on making two sequential choices. The ability to plan across the two choices using structural knowledge of the task (“model-based”) can be dissociated from simple repetition of actions which lead to reward (“model-free”). This is because the task exploits a probabilistic structure where the majority of trials consist of predictable chains of events (common trials), but in a subset of trials, the two chains of events are swapped over (rare trials). A decision-maker who uses a model-free strategy will be more likely to repeat an initial choice leading to reward, regardless of whether the previous trial followed a common or rare sequence of events (main effect of outcome on stay probability). However, a decision-maker who uses a model of the task structure will be more likely to repeat their first step choice after being rewarded on a common trial, but switch to the opposite choice when rewarded on a rare trial (outcome–transition interaction on stay probability).

Notably, across our population of older subjects, the use of model-based strategies was attenuated. While participants (pooled across groups) were more likely to stay after being rewarded (main effect of reward; two-sided one-sample Wilcoxon: $\bar{\beta }=0.57$, *n* = 69, *Z* = 4.48, *p *= 7.63 × 10^−6^), they did not modulate their behavior significantly depending on the rarity of the transition experienced (Fig. 3*e*,*f*; outcome–transition interaction; two-sided one-sample Wilcoxon: $\bar{\beta }=0.17$, *n *= 69, *Z* = 0.90, *p *= 0.368). This is consistent with previous studies that found reduced model-based reasoning in older populations (Eppinger et al., 2013). Importantly, we also did not find any difference between groups in either model-free or model-based strategies as a function of brain damage (Kruskal–Wallis test for effect of lesion group on main effect of reward: *H*_(2)_ = 0.56, *p *= 0.754; Kruskal–Wallis test for effect of lesion group on outcome–transition interaction: *H*_(2)_ = 1.56, *p *= 0.458; Fig. 3*f*).

Despite this, all groups were sensitive to the transition structure linking Step 1 and Step 2 as indicated by slower response times following the more surprising rare transition compared with the common transition (Fig. 3*d*; Wilcoxon signed-rank of mean response times after rare vs common transitions; vmPFC: *Z *= 4.68, *n *= 30, *p *= 2.88 × 10^−6^; LCs: *Z *= 3.22, *n *= 19, *p *= 0.001; HCs: *Z *= 3.21, *n *= 20, *p *= 0.001). Again, we did not find that lesion damage significantly affected sensitivity to the task structure as reflected in response times (Kruskal–Wallis for effect of lesion group on response time difference following common vs rare transitions: *H*_(2)_ = 2.56, *p *= 0.279).

Finally, we fit reinforcement learning models to the data to formally quantify the contribution of model-based and model-free reasoning, while also controlling for other behavioral factors such as persistence (the tendency to repeat the previous action) and learning rate (how quickly people update their beliefs in the value). We found no significant group differences in model-based behavior when formally modeled (Fig. 3*g*; Kruskal–Wallis for effect of lesion group on model-based *β* weight in RL model: *H*_(2)_ = 4.73, *p *= 0.094).

While our hypothesis that model-based planning would be lower in vmPFC patients was not supported by the data, in theory, vmPFC damage could affect behavior through other parameters in the reinforcement learning model. Since we had no other hypotheses regarding the remaining parameters, we performed exploratory analyses to determine if there was an effect of lesion group on these metrics. None of the five remaining parameters from the RL model showed any significant differences as a result of group (Kruskal–Wallis tests; model-free weight: *H*_(2)_ = 0.65, *p *= 0.722; shown in Fig. 3*h*; Step 2 *β*: *H*_(2)_ = 0.51, *p *= 0.777; persistence bias: *H*_(2)_ = 2.00, *p *= 0.367; *α*: *H*_(2)_ = 1.70, *p *= 0.428; *λ*: *H*_(2)_ = 0.173, *p *= 0.917). Our findings suggest that across a range of behavioral metrics, vmPFC patients neither demonstrated significantly worse performance nor showed significant differences in reinforcement learning in the Two-Step task.

## Discussion

Damage to vmPFC has long been associated with differences in planning in complex environments, but how this relates to the underlying cognitive processes supporting planning is unclear. We investigated how vmPFC damage affects complex planning using a recently developed task and computational framework called “Four-in-a-Row,” which enables the dissociation of multiple components of planning (van Opheusden et al., 2023). Consistent with findings from more naturalistic settings, vmPFC damage was associated with worse performance in this rich multistep planning game compared with lesion control patients and age-matched healthy controls. We investigated how planning deficits related to the cognitive processes identified by a planning model, through three possibilities: that vmPFC damage leads to more feature oversights, to reductions in planning depth, or to systematic deviations in how options are heuristically evaluated. We found the first two of these hypotheses to be true. Patients with vmPFC damage were more likely to overlook game-relevant features on the board, leading to missed opportunities for winning or blocking opponents. vmPFC damage was also associated with planning less far into the future compared with patients with damage to other brain areas.

We investigated whether planning differences revealed using Four-in-a-Row could be detected in another planning paradigm with a substantially smaller set of options and a shorter planning horizon. Four-in-a-Row characterizes planning over multiple steps and a vast number of candidate options, making it more similar to real-world planning than many previous laboratory planning tasks (van Opheusden et al., 2023). In contrast, the “Two-Step” task probes decision-making at a maximum horizon of two steps into the future, with only two options available at each step. In this setting, we did not find any behavioral differences between the three groups.

However, the Two-Step findings in our study are limited by the fact that no group demonstrated significant model-based planning. This is likely to be related to the age of the population, where older cohorts generally show reduced metrics of model-based behavior (Eppinger et al., 2013). Importantly, various studies have shown that model-based strategies in the Two-Step paradigm do not achieve substantially greater reward than simpler model-free strategies. This could mean that subjects who have the capacity to deploy model-based strategies are insufficiently incentivized to do so in this paradigm, instead relying on less effortful model-free strategies (Kool et al., 2016, 2017). However, the fact that we found no difference in Two-Step behavior across lesion groups is unlikely to be explained by a lack of power, as our sample size for the Two-Step task was substantially larger than for most lesion patient studies (Yu et al., 2020), including studies detecting location-based differences using the Two-Step paradigm (Vikbladh et al., 2019).

While the Two-Step task failed to reveal evidence of planning in this older population, the Four-in-a-Row task was able to detect strong signatures of planning as well as behavioral differences between groups, even with a smaller sample size. This suggests that the Four-in-a-Row task provides a more sensitive computational framework for studying planning that can be used even in older populations, whereas the Two-Step task loses sensitivity for detecting planning behavior. Since the Two-Step task and Four-in-a-Row differ on many dimensions, it is not possible to determine the main factors driving the difference in sensitivity between these tasks. On the one hand, it is possible that the tasks measure the same cognitive processes but that the larger state space involved in playing Four-in-a-Row enables greater sensitivity for detecting subtle behavioral differences. On the other hand, it is possible that the differences observed between paradigms arise from their dependency on diverging cognitive constructs. For example, the Two-Step task requires accessing unobservable knowledge of the transition structure and having an understanding of the stochasticity of state transitions, while Four-in-a-Row requires simulating the moves of an opponent player. Finally, as discussed earlier, another possibility is that participants were simply not motivated to deploy model-based planning in the Two-Step task (as opposed to Four-in-a-Row), which could be ameliorated by adapting the Two-Step task to incentivize model-based strategies (Kool et al., 2016, 2017). An important avenue for future investigation will be to study the relationship between parameters in these two planning tasks among the wider healthy population, to understand the cognitive components supporting task performance in both settings.

When choices in the Four-in-a-Row task were investigated using a computational planning model, we found that vmPFC patients were more likely to overlook game-relevant information on any given trial (“feature drop”). This metric captures the tendency to miss critical features on the game board, for example, opportunities for winning or blocking opponents. One possible cause for this behavior is that vmPFC damage affects the ability to integrate all relevant information for making decisions. This is consistent with previous work showing that patients with vmPFC damage fail to make decisions which require integrating multiple value-relevant attributes (Fellows, 2006; Pelletier and Fellows, 2019). Another possibility is that patients failed to orient attention to value-relevant features of the environment. This idea is also consistent with previous studies showing vmPFC damage alters the allocation of attention to valuable features of the environment (Vaidya and Fellows, 2015), while activity in vmPFC predicts goal-oriented attention in healthy individuals (Günseli and Aly, 2020; Holton et al., 2024). Future work could disentangle these different mechanisms leading to more feature oversights, for example, by using eye-tracking.

As well as overlooking game-relevant features, we found that vmPFC patients planned to lower depth than control patients. However, in contrast to the previous finding relating vmPFC damage to feature oversights, this finding did not survive comparison with a healthy age-matched population. Planning involves a mental simulation of possible futures that one could encounter. Problems simulating the future will lead to suboptimal planning and has been one of the proposed explanations for planning deficits in vmPFC patients (Bertossi et al., 2017). As far as we know, this finding that vmPFC patients planned to lower depth than control lesion patients is the first evidence from a computational paradigm to support a wealth of qualitative evidence suggesting vmPFC patients have difficulties producing details about the future (Fellows and Farah, 2005; Bertossi et al., 2016a,b, 2017). Difficulty in planning deeply may arise due to damage to an internal map of the causal task structure, which has been localized to vmPFC in neuroimaging (Wilson et al., 2014; Schuck et al., 2016). Alternatively, future planning may be attenuated if the temporal discount factor is very steep (i.e., decisions are dominated by proximal rewards), as is sometimes observed after vmPFC lesions (Sellitto et al., 2010; Peters and D'Esposito, 2016; although note that results have been conflicting; Fellows and Farah, 2005).

While we found that vmPFC damage reduces planning depth, the simulation of future states is likely to rest on distributed neural mechanisms. One possibility is that vmPFC is critical for coordinating task-dependent computations performed in other areas such as the hippocampus or striatum (Blanco-Pozo et al., 2024). Consistent with this idea, damage to the hippocampus has been shown to impair model-based planning in rodents (Miller et al., 2017) and most recently in humans performing the Two-Step task (Vikbladh et al., 2019). Greater functional coupling between the hippocampus and vmPFC has also been shown to predict better inferences over unseen structured relationships in healthy individuals (Zeithamova et al., 2012), and many tasks involving model-based inferences find activity in both the vmPFC and hippocampus (Barron et al., 2013, 2020; Redish, 2016; Wang et al., 2020; Park et al., 2021), supporting the possibility that prefrontal areas may coordinate model-based simulation played out in hippocampal areas.

There is growing evidence that people use heuristic strategies to plan when the environment is too complex to simulate all possible future (Huys et al., 2012, 2015; Solway et al., 2014; Solway and Botvinick, 2015; Snider et al., 2015; Keramati et al., 2016; van Opheusden et al., 2023). We did not find evidence that vmPFC patients were significantly worse at identifying what constituted a good heuristic for evaluating moves compared with controls (“heuristic quality”). This alternative metric captured the difference between how participants weighed up different heuristic features for evaluating candidate moves compared with an optimal player.

Although controlling for education and age did not alter our results, the substantially lower education levels in the vmPFC cohort and the variability in age across the different control groups remain a limitation of our study. Future studies could address these issues through more precise participant matching.

In conclusion, we leveraged recent computational methods for studying planning in patients with frontal brain lesions. The rich framework of the Four-in-a-Row task revealed that deficits in complex planning following vmPFC damage are related to tendencies to overlook relevant information and to plan less deeply into the future. This contrasted with a simpler paradigm for studying planning, namely, the Two-Step task, which failed to reveal behavioral differences between groups. Novel computational methods for capturing behavior in rich task settings offer exciting new opportunities for meeting the age-old challenge of balancing complexity and interpretability in lesion patient studies.

## Data Availability

The processed data will be made publicly available upon publication at https://osf.io/4xm86/, in addition to the custom code for reproducing the manuscript figures and analyzing the Two-Step data. The custom code for analyzing the Four-in-a-Row data can be found at https://github.com/basvanopheusden/fourinarow.
